# *DUSP5* and *DUSP6* modulate corneal epithelial cell proliferation

**Published:** 2010-08-22

**Authors:** Zheng Wang, Peter S. Reinach, Fan Zhang, Kati-Sisko Vellonen, Arto Urtti, Helen Turner, J. Mario Wolosin

**Affiliations:** 1 Department of Biological Sciences, SUNY State College of Optometry, New York, NY; 2 Centre for Drug Research, University of Helsinki, Helsinki, Finland; 3 Department of Ophthalmology and the Black Family Stem cell Institute, Mount Sinai School of Medicine, New York, NY

## Abstract

**Purpose:**

Dual specificity phosphatases (DUSPs) modulate the duration and magnitude of phospho-activation of Erk1/2, p38 and JNK1/2, the terminal kinases (TKs) of the mitogen activated protein kinase  (MAPK) cascades. Three DUSPs, DUSP1, DUSP5, and DUSP6, are overexpressed in ocular surface side population stem cells (SPSCs). Our objective was to identify the impact of these enzymes on TK phosphorylation and proliferation of corneal epithelial cells.

**Methods:**

SV40 immortalized (sv) and expanded fresh human corneal epithelial cells (efHCECs) were transduced with lentivectors to elicit expression of shRNAmir against *DUSP1*, *DUSP5*, and *JNK1* to thereby create the DUSP1i, DUSP5i and JNKi cell sublines, or overexpress DUSP6 (henceforth DUSP6^+^), respectively. TK phosphorylation status and proliferation rates were determined by immunoblotting and ^3^H thymidine uptake.

**Results:**

In both ef and svHCECs, EGF supplementation after a 24 h serum starvation caused a rapid 5–15 min spike in the phosphorylation of all three TK types. This was followed by gradual decreases to low phosphorylation levels within one h. These declines coincided with dramatic increases in DUSP1 and DUSP5 protein expression. In DUSP1i, the DUSP1 increase was abolished. All 3 TKs maintained high phosphorylation levels for at least 90 min and proliferation rates were unchanged from non-transduced cells. In DUSP5i, the DUSP5 protein increase was prevented, the post peak phosphorylation decrease occurred only on Erk1/2 and the proliferation rate increased by 50%–60%. In JNK1i, JNK1 was essentially knocked out and proliferation rates were also markedly elevated. At steady-state, DUSP1i maintained high levels of pJNK1/2 expression. In DUSP6^+^ Erk1/2 phosphorylation was prevented and proliferation rates decreased to less than 50%.

**Conclusions:**

DUSP5 and DUSP6 selectively control ERK pathway activity and proliferation. The lack of an effect of DUSP1 knockdown on proliferation can be attributed to its pan-MAPK effect. The expected augmented proliferative response due to enhanced and prolonged phosphorylation of Erk1/2 following DUSP1 knockdown does not occur because a pJNK1/2 antiproliferative effect is simultaneously unleashed.

## Introduction

Mitogen activated protein kinase (MAPK) cassettes are a super family composed of signaling pathways that transduce different extracellular signals to elicit a host of cell specific responses. Erk1/2, p38 and JNK1/2 are terminal kinases (TKs) in these pathways. These enzymes phosphorylate numerous substrates, including cytosolic and nuclear transcription factors [1-4]. Nuclear transcription factor activation occurs as consequence of rapid TK translocation from the cytoplasm to the nucleus [5-7]. The Erk1/2 pathway is intimately associated with the control of growth factor activated cell proliferation primarily as a result of its effect on transcription factors that facilitate the G_1_ to S cell cycle step [5]. The phosphorylation of the TKs is modulated by a family of TK-targeting (typical) dual specificity phosphatases (DUSPs). DUSPs can simultaneously dephosphorylate MAPK serine and tyrosine residues. There are 10 typical DUSPs enzymes designated DUSP1, DUSP3 through DUSP10, and DUSP16 [8-10]. Each one of these enzymes displays distinct features in their MAPK specificity, cellular localization and responsiveness to cellular activation. DUSP5 is a vaccinia virus-related, Erk1/2-specific inducible nuclear enzyme that is rapidly induced following MAPK activation [11], DUSP1, while nuclear and inducible, shows a pan-MAPK activity spectrum [12] and DUSP6 while substantially selective for Erk1/2, has a cytosolic location [13]. Many of the reported features of the DUSPs are based on results obtained in vitro in which purified DUSPs were used to characterize their specific interactions with MAPKs. In practice, expression profiles, compartmentalization, responsiveness to cellular activation and MAPK-DUSP stoichiometry may all contribute to the modulation of MAPK controlled activities in each particular cell lineage or condition.

We have recently shown that ocular surface epithelial stem cells (SPSC) possessing the quiescent, slow cycling phenotype [14-16], display high constitutive expression levels of several typical DUSPs, including DUSP1, DUSP5, and DUSP6 [17,18]. Given the aforementioned DUSP features, we proposed that this overexpression contributes to the slow cycling features of adult SPSC [17,18]. In this report, using lentiviral transduction methodologies to impose changes in DUSP expression levels, we show that increased DUSP5 and DUSP6 expressions are likely to be factors in the slow cycling phenotype of SPSC through their effect on dephosphorylating Erk1/2 whereas DUSP1 may instead primarily control other functions such as innate immune responses to stress via modulation of JNK1/2 activation.

## Methods

### Cell systems

Human SV40 immortalized corneal epithelial cells (svHCECs) were a generous gift from Dr. Araki Sasaki (Ideta Eye Hospital, Kumamoto City, Kumamoto, Japan). These cells were cultured in a 1:1 mix of Dulbecco’s modified medium and Ham F12 (D/F12; Invitrogen, Carlsbad, CA) complemented with 10% FBS (Invitrogen) and 5-ng/ml epidermal growth factor (EGF). Expanded fresh human corneal epithelial cells (efHCECs) were isolated by the following procedure. Three explants of corneal limbal-peripheral segments dissected from unidentified cadaver corneas provided by the National Disease Research Interchange (Philadelphia, PA) were cultured for 10 days in SHEM (a 95.0: 5.0:0.5 mix of D/F12, FBS, and DMSO complemented, per liter, with 10 μg each of insulin, transferrin, and selenium, 10 ng cholera toxin, 5 ng EGF, and 28 mg phosphoethanolamine) and for 2 more days in EpiLife (Invitrogen), a low calcium Boyce and Ham type formulation. The outgrowths were trypsinized and expanded in culture for 12 generations in EpiLife before study. The corneal identity of these cells was confirmed by showing that raising medium calcium to 1 mM induced keratin 3 expression [19].

### Lentivectors

Lentivectors for stable expression of shRNAmir sequences against *DUSP1*, *DUSP5*, and *JNK1* were generated using pGIPz plasmids clones V3LHS_352110, V2LHS_61931, and V3LHS_170502, respectively (Open Biosystems, Hunstville, Al). Blast analysis of the 20-mer antisense sequences expressed by these clones demonstrated that in each case only the intended target RNA was a full sequence match. The expression cassette incorporated into the host cell DNA by these lentivectors drives the expression of a turbo-GFP protein, the shRNAmir sequence and a puro^r^ gene from a single CMV promoter. Stable overexpression of *DUSP6* open reading frames (ORF) was accomplished using lentivectors based on the pLEX plasmid (Open Biosystems). The pLEX cassette drives expression of a MYC tagged ORF and the puro^r^ gene.

Viral particles were generated by transducing HEK293T cells cultured in 100 mm dishes with 2 μg active plasmid, 8 μg of packing plasmid mix (UMIX^™^, Rockville, Md.) in 10 ml medium (DMEM with 10% FBS) containing 20 μl HEKFectin (BioRad, Richmond, CA). The culture medium was refreshed after overnight incubation and virus-rich supernatant was collected 48 h later after confirming that most 293 cells developed strong GFP fluorescence. The medium was then exchanged with EpiLife and simultaneously concentrated sixfold using Amicon Ultra 100,000 daltons cutoff filters (Millipore, Billerica, MA). Transductions with fractions of the purified vector suspensions were performed in semi confluent 6-well dishes in EpiLife. Transduced cells were selected with puromycin (15 μg/ml) using the disappearance of all GFP negative cells and elimination of all cells from an untransduced control culture as selection end points.

To generate a control subline for non-specific effects of shRNAs, we purchased viral particles incorporating a cassette for the simultaneous expression of a non-coding shRNA and puromycin resistance (Sigma, St Louis, Mo). After puromycin selection, these cells were used to determine any potential non-specific effect of the expression on cell properties, in particular on the TKs.

### Flow cytometry

Forward (FSC) and side (SSC) scatter flow cytometry was used to determine any putative changes in the relative size or intracellular complexity of the cells, respectively, induced by the expression perturbation. The complete elimination of untransduced cells and degree of GFP expression in the puromycin pGIPz transduced cells was also determined by flow cytometry. Measurements were made in an Accuri 6 (Ann Arbor, MI) flow cytometer.

### Immunostaining

Control and puromycin selected cells transduced with the pLEX-DUSP6 ORF were immunostained with a FITC-conjugated anti MYC tag antibody (Millipore).

### Western blotting

Cultured cells were lysed, sonicated, and spun down. The supernatant was collected, protein concentration was measured with the BCA^™^ protein assay kit (Thermo Scientific, Rockford, Il) and identical protein amounts were electrophoresed in 10-well 10% polyacrylamide gels, electroblotted to nitrocellulose membranes, and reacted with primary antibodies. After proper washings, the membranes were reacted with the appropriate HRP-conjugated antibody. HRP levels were determined by chemilumiscence using ECL (General Electric, Buckinghamshire, UK). Antibodies for Erk1/2, pErk1/2, were purchased from Santa Cruz (Santa Cruz, CA); p38, p-p38, JNK/SAP and p-JNK/SAP were from Cell Signaling, DUSP1 and DUSP5 were from Abnova (Walnut Creek, CA). Relative HPR signal intensities were manually determined with the histogram feature of Photoshop using in each case the signal free proximal area to subtract a background value.

### Tritriated thymidine uptake

Triplicate cultures were incubated at 37 °C for 2 h with 1 μCi/ml [3H]thymidine (3.3 to 4.8 TBq/mmol). They were then washed twice with cold PBS, three times with ice-cold 5% trichloroacetic acid (TCA), and twice with cold 90% alcohol. Cell lysis was obtained with 0.2 N NaOH/0.2% SDS. Radioactivity was measured in a Tri-Carb 2900TR instrument (Perkin-Elmer, Boston, MA) and the data were normalized to cellular protein content determined with a BCA protein assay kit.

### Growth curves

Twenty thousand control and subline cells were seeded on 6-well plates and cultured for 96, 120, and 144 h. At the indicated times, duplicate cultures were trypsinized using 950 μl enzyme and a 20 min incubation, to ensure complete cell-substratum and cell-cell dissociation. The single cells suspensions were supplemented with 50 μl FBS, and the amount of cells in 100 μl suspension, which in all cases exceeded 10,000 cells, was accurately determined using the cell counting facility of the Accuri instrument.

### Gene expression of typical DUSPs in corneal epithelium and svHCECs

MAS 5 (Affymetrix, Santa Clara, CA) signal intensities and present/absent status for 9 typical DUSPs were obtained from the Affymetrix 95 A chip-based gene expression studies on intact human corneal epithelium from cadaver donor corneas by Turner et al. [20]. The data are available through the accession number GSE5543 at the NCBI Gene Expression Omnibus (GEO). The same U95 A microarray has been used to characterize global gene expression in stratified epithelia generated using the svHCECs. Briefly, svHCECs were cultured on collagen-coated polyester filters (Transwell-Clear™; Costar, Cambridge, MA) in medium supplemented with 40 mg/ml L(+)-ascorbic acid at the liquid–air interface [21]. Stratified cultures with transepithelial resistance values greater than 400 ohm/cm^2^ were dissolved in TriReagent^™^, total RNA was isolated, processed for microarray hybridization and results were extracted from the raw hybridization images using MAS5, as described [20]. A complete description of all the results of this latter study besides those related to *DUSP* expression, will be presented elsewhere.

## Results

The analysis of the microarray data for fresh tissue corneal epithelium shows that of nine DUSPs represented in the array (*DUSP16* is not represented), only four, *DUSP1*, *DUSP5*, *DUSP6*, and *DUSP10* were appreciably expressed (Table 1). The validity of the MAS5 SI readings for these DUSPs was corroborated by real time PCR in human conjunctival epithelial cells [17]. The other five either had signal intensities barely above background and/or partially or completely failed the present/absent MAS 5 test. Comparable results were obtained in a similar U95A-based gene expression study using the svHCEC cell line after the cells had undergone stratification at an air water interface. The sv transformed cells though had an additional moderate expression of *DUSP2* and *DUSP4* (Table 1). This is consistent with the genomic aberrations observed in these cells [22].

**Table 1:** Expression of typical DUSPs in whole corneal epithelium derived from fresh tissue and in pseudo-epithelium generated in culture by svHCEC cells.

		**DUSP**									
**Sample**	**Parameter**	**1**	**2**	**4**	**5**	**6**	**7**	**8**	**9**	**10**	**16**
Tissue	SI	7,091.6	30.3	17.9	325.1	394.9	46.7	35.1	33.2	84.1	NR
	P/A/M	PPP	AAA	AAA	PPP	PPP	PAA	PAA	PPA	PPP	
svHCEC	SI	5,512.4	150.1	235.9	392.8	138.8	11.7	19.2	36.8	129.2	
	P/A/M	PPP	PPA	PPP	PPP	PPP	AAA	AAA	PAM	PPP	

Signal intensities (SIs) represent the average of three independent triplicates in each case. The Present/Absent/Marginal MAS 5 readings (P/A/M) for each triplicate are presented in concatenated format. NR, not represented in the array.

**Figure 1:**
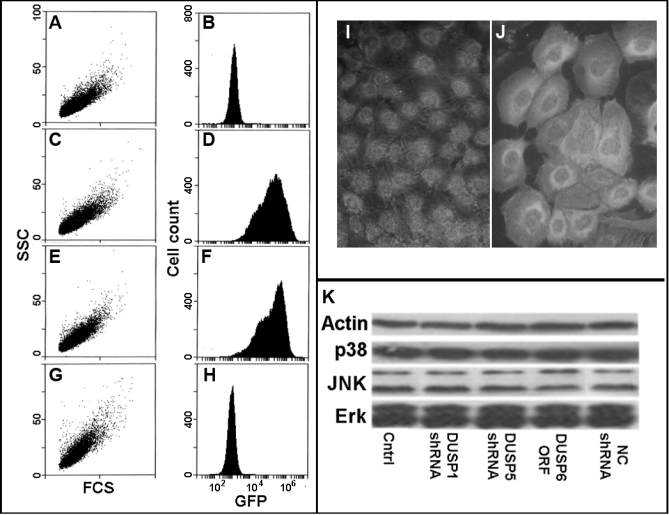
Basic features of svHCECs transduced with various lentivectors. **A-H**: Light scatter plots (**A**, **C**, **E**, and **G**) and histograms of GFP fluorescence (**B**, **D**, **F**, and **H**). **A**,**B**: control cells; **C**,**D**: *DUSP1* shRNA cells; **E**,**F**: *DUSP5* shRNA; **G**,**H**: *DUSP6* ORF. **I**,**J**: Control cells (**I**) and cells transduced with the MYC tagged *DUSP6* ORF (**J**). In the control stain is seen exclusively in the nucleus, corresponding to the location of myc expression. In the transduced cells there is additional stain throughout the cytosol including strong stain in the perinuclear area. **K**: western blot of all three terminal MAPKs and actin for the control cells and cells transduced with *DUSP1*, *DUSP5*, and non coding (NC) shRNA and the MYC tagged *DUSP6* ORF.

Figure 1 depicts comparative features of the control svHCEC and the sublines generated by lentiviral transductions. Flow cytometry showed that neither the forward light scatter nor the side light scatter, relative measures of cell size and granularity, respectively, were affected by the combined expressions of GFP, shRNA and puro’ induced by the pGIPz vector (Figure 1A,C,E). The expression of DUSP6 caused a 12% increase in cellular granularity. The GFP levels attained with the pGIPz lentivectors for DUSP1 and DUSP5, a direct measure of the expression of the shRNAmirs were comparable (Figure 1D,F). Since the pLEX vector for DUSP6 expression does not express a fluorescent protein (Figure 1H) the expression was determined using an anti MYC tag antibody (Figure 1I,J). In all cells, the tag was visible and concentrated in the perinuclear (i.e., ribosomal) zone, suggesting active synthesis, and evenly distributed throughout the cytosol. In addition, we determined by western blotting that neither of the applied transductions affected the levels of expression of the three TKs or of β-actin (Figure 1K). Since the amount of actin per cell is usually considered invariant, this latter result implies a constant amount of protein per cell.

**Figure 2:**
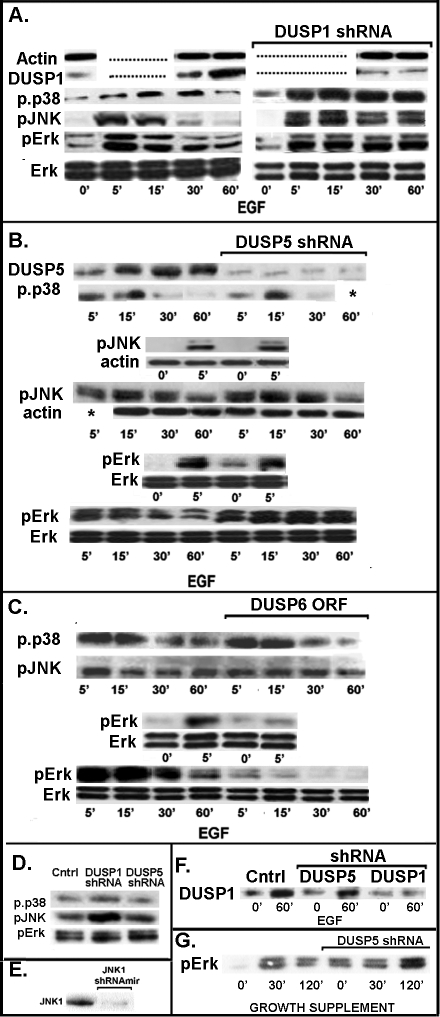
Effects of *DUSP1*, *DUSP5*, and *DUSP6* expression changes on the steady-state and post activation time course of terminal kinase phosphorylation in svHCEC and efHCECs cells. **A**: Effect of *DUSP1* shRNAmir. The antisense agent prevented the post EGF activation increases in DUSP1 protein and concomitantly abolished or diminished the decline in the initial EGF induced phosphorylations of Erk1/2, JNK1/2, and p38 normally occurring in the control. **B.** Effect of *DUSP5* shRNAmir. The antisense agent completely blocked the post-EGF increase in DUSP5 protein levels and abolished the post peak decline in pErk1/2 phosphorylation. This agent did not visibly modify the time course of the phosphorylation responses of JNK1/2 or p38. **C.** Effects of overexpression of *DUSP6*. Phosphorylation of Erk1/2 in response to EGF was virtually abolished. The effects on the other two terminal kinases were nil or minimal. **D.** Comparison of the effects of shRNAmirs against *DUSP1* and *DUSP5* expression on the phosphorylation of the terminal kinases in cells growing in log phase (50%–80% confluent) in whole culture medium. Both antisense interventions increased pErk1/2 steady-state levels. DUSP1 selectively increased pJNK1/2 phosphorylation. Neither had an effect on p-p38 steady-state expression. **E.** Effect of expression of *JNK1* shRNAmir on the expression of JNK1 protein in svHCEC cells. **F.** Experiment showing that the permanent transduction with *DUSP5* shRNA does not interfere with the rise in DUSP1 protein that occurs after cell reactivation with EGF. **G.** Effect of *DUSP5* shRNAmir on pErk1/2 phosphorylation in the efHCECs cells. Blots **A**-**E** are representative of two or more repeats.

Figure 2 summarizes the effects of the lentivector-induced modification of DUSP activity on the phosphorylation levels of all three TKs in the svHCEC. Unless indicated otherwise, cells were starved of growth factors by incubating them for 24 h in base media (D/F12 for svHCECs, complement-free EpiLife for the efHCECs using multiple replicate cultures. Next, cells were reactivated by the addition of either 10 ng/ml EGF in the case of the svHCEC or EpiLife complement (contains bovine pituitary extract and insulin) in the case of the efHCECs. Cultures were collected just before reactivation or 5, 15, 30, and 60 min thereafter. For the *DUSP1* shRNA experiments, the five control and five transduced cell time points were run side by side, each with MW markers. For the *DUSP5* shRNA and the *DUSP6* ORF, to allow the side by side time course comparison to be present in a single 10-well gel, we ran separately one gel for the 0–5 min time points and another for the 5–60 min comparisons.

In the untransduced svHCEC, addition of EGF caused a rapid and dramatic increase in the phosphorylation status of all three TK families within 5 to 15 min, as previously described [23]. In all cases, this was followed within the next 60–90 min by a return toward substantially lower steady-state levels (Figure 2A-C). These decreases coincide with rises in DUSP1 and DUSP5 protein concentration. Cells transduced with the non-coding shRNA showed identical responses (not shown).

**Figure 3:**
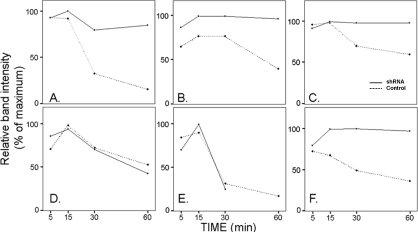
Densitometric plots of the phosphorylated MAPK bands displayed in Figure 2. **A** and **D**: p.p38. **B** and **E**: pJNK1. **C** and **E**: pErk1. Top frames are for *DUSP1* shRNA, bottom frames for *DUSP5* shRNA. The values were normalized respective to loading controls, either total Erk or actin.

In the cells transduced with anti DUSP1 shRNAmir, the increases in DUSP1 were essentially abolished and all three TKs maintained high levels of activity throughout the first 60 min of post-EGF exposure. In the cells transduced with the DUSP5 shRNAmir, the DUSP5 rise was nullified and Erk1/2 continuously increased over this period, but the phosphorylation changes in the JNK/SAPK and p38 kinases were not affected. The differences in phosphorylation in response to EGF between the cells transduced with shRNAs for DUSP1 and DUSP5 are shown in Figure 3 in densitometric plots of the results presented in Figure 2A,B.

DUSP6 overexpression specifically prevented the formation of any substantial levels of pErk1/2 (Figure 2C). The EGF-induced phosphorylation of p38 and JNK1/2 did not seem to be affected by DUSP6 overexpression; small effects on JNK1/2 were observed in a second identical test (not shown).

Next, we examined in a comparative manner the steady-state levels of phosphorylation of all the TKs in cells not subjected to the starvation and reactivation cycle. Inhibition of either *DUSP1* or *DUSP5* by their respective shRNAmir vectors caused similar, moderate increases in pErk1/2. In addition, the *DUSP1* anti-sense selectively caused a strong enhancement of pJNK1/2 formation (Figure 2D). Neither the *DUSP1* nor the *DUSP5* shRNAs caused a substantial change in p-p38 relative to control in this condition. Measurements of *DUSP1* expression before and 60 min after addition of EGF showed that the *DUSP5* shRNA did not interfere with the control rise in *DUSP1* described in Figure 1A-H (Figure 2F). The effects of changes in DUSP expression on Erk1/2 phosphorylation were also determined in efHCECs that were maintained in the low calcium ([Ca^2+^]=0.06 mM), serum-free EpiLife medium to prevent differentiation [24]. Growth factor deprivation followed by reintroduction of the EpiLife growth factor supplement caused changes in Erk1/2 phosphorylation similar to those observed with the svHCECs (Figure 2G). Intriguingly, the comparison of 0 times for both svHCEC and efHCEC suggested that the *DUSP5* shRNA was uniquely able to increase the pErk1/2 levels even in growth factor-free conditions (Figure 2B and Figure 2G).

**Figure 4:**
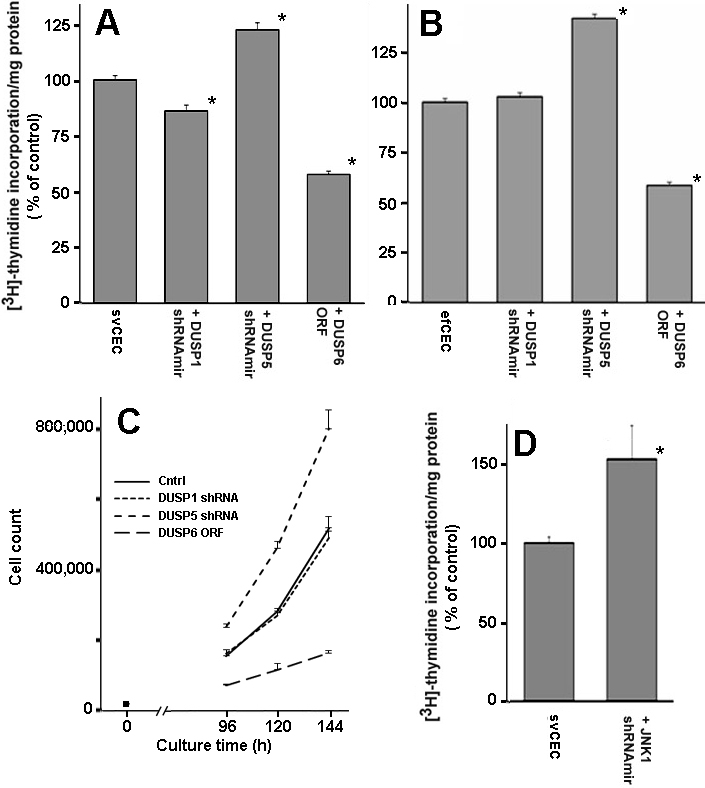
Relative thymidine incorporation and proliferation rates of svHCEC and efHCECs transduced with *DUSP1* or 5 shRNAmirs or the *DUSP6* ORF in the presence of 10 ng/ml EGF. **A**: Effects of DUSP transductions on svHCECs. **B**: Effects of DUSPs transductions efHCECs. **C**: Growth curves for the control svHCEC and the transduced cell lines. **D**: Effect of *JNK1* shRNAmir on proliferation of svHCECs. Thymidine uptake data represents the mean±SEM of three independent experiments. The asterisks indicate p<0.01. Growth curves shown are for a single experiment representative of two independent measurements. Bars are the average deviation for the duplicates in the experiment shown.

Finally, we correlated the changes in TK phosphorylation patterns with thymidine uptake and proliferation rates (Figure 4). In both svHCEC (Figure 4A) and efHCEC (Figure 4B) cultures, *DUSP5* shRNAmir and *DUSP6* ectopic gene expression, respectively, augmented and depressed thymidine uptake (p<0.01). The *DUSP1* shRNAmir, in contrast, caused a statistically significant small decrease in uptake rate in the svHCEC and had no statistically significant effect in the efHCEC. Growth curves for the svHCEC cells confirmed that the changes in thymidine uptake reflected changes in proliferation rates (Figure 4C). The *DUSP5* shRNA and the *DUSP6* ORF cell lines grew faster and slower, respectively, than either the untransduced control or the *DUSP1* shRNA subline. From the changes in cell numbers between two successive time points, we calculated doubling times of 26, 26, 21, and 45 h for the untransduced control, *DUSP1* shRNA, *DUSP5* shRNA, and *DUSP6* ORF cell lines, respectively.

The combined results of Figure 2 and Figure 4 for the svHCEC cells demonstrated that while *DUSP1* and *DUSP5* knockdown enhanced Erk1/2 phosphorylation to similar levels, only the latter caused a substantial change in proliferation. Hence, since the immunoblots shown in Figure 2D indicated that the main difference between the effects of *DUSP1* and *DUSP5* shRNAmirs were on the steady-state levels of activated JNK1/2, we investigated if the changes in these enzyme levels could be a factor in the overall proliferation rate; we found that drastic reduction of JNK1 expression by its shRNAmir lentivector (Figure 2E) was sufficient to cause a substantial increase in proliferation rate (Figure 4D).

## Discussion

SPSCs are in a slow cycling state in vivo [16] and display poor clonogenic capacity [16,25]. We have proposed that this latter feature reflects their inability to undergo rapid proliferation when suddenly transferred from the in vivo to the artificial ex vivo condition. In support of this notion, a) bone marrow SP cells similarly fail to initiate colonies immediately after extraction, yet convert into highly clonogenic cells following a period of organ culture in a semi in vivo environment [26] and b) similarly change from low to high clonogenicity when limbal segments were cultured in vitro for a week before single limbal epithelial cell collection (unpublished).

**Figure 5:**
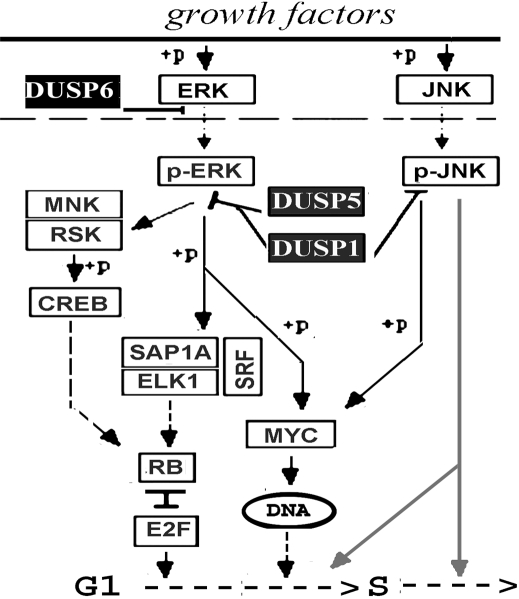
Schematic description of proposed nuclear events associated with the transductions performed in this study. Erk1/2 phosphorylation causes nuclear translocation and activation of transcription factors that facilitate the G_1_ to S transition. JNK phosphorylation causes nuclear translocation and activation of antiproliferative events at some stage of the cell cycle (indicated by gray lines). The proliferation-enhancing event is blocked by pErk/2 dephosphorylation, by DUSP6 in the cytosol and DUSP5 and DUSP1 in the nuclei. The latter DUSP though, blocks JNK1/2 phosphorylation with high efficiency nullifying its pro-proliferative action.

Gene microarray studies of SP stem cells from pig limbus [18] and human conjunctiva [17] and limbus (unpublished) revealed several features that have the potential to underpin this slow cycling phenotype. One of them is the high expression level of several DUSPs, relative to those of their nonSP counterparts. *DUSP5* is the most prominently overexpressed DUSP in the two published studies. It is 10.6 times higher in the human conjunctival SP cell [17] than in the nonSP counterparts, 2.8 times better expressed in the pig limbus [18] and 21 times more in the human limbus (unpublished), respectively. *DUSP1* is also overexpressed in these systems, but to a lesser extent. *DUSP6* is overexpressed in the human system, but not in the porcine cells. Additionally, *DUSP1* gene expression levels are intrinsically very high, similar to those of housekeeping enzymes such as β-actin (*ACTB*) or *GAPDH* [17]. These observations make it unlikely that the other more weakly expressed DUSPs are significant players in the regulation of MAPK dependent cell activities. In this context, the current results demonstrate that, a) DUSP5 affects the phosphorylation state of Erk1/2 in the presence of unmodified DUSP1 activity; b), sustained phosphorylation of Erk1/2 induced by inhibition of DUSP5 activity is associated with increased epithelial cell proliferation; and c) conversely, prevention of Erk1/2 phosphorylation by DUSP6, causes a major decrease in proliferation rates. In contrast to these seemingly straightforward relationships between Erk1/2 phosphorylation and proliferation rates, no measurable change in proliferation rates were seen when the Erk1/2 levels were augmented via *DUSP1* shRNAmir vector, in either the sv or efHCEC systems. This latter result may be related to an intrinsic anti-proliferative feature of activated JNK1/2 in these cells, as suggested by the enhanced thymidine uptake rate observed in the JNK1 deficient sub line (Figure 4D). Thus, even though DUSP1 knockdown prolonged and enhanced Erk1/2 phosphorylation, its concomitant elevation of pJNK1/2 levels may nullify the pro-proliferative effect of pErk1/2, suggesting the existence of set points for protein concentrations, affinities and activity rates intrinsically calibrated to buffer the pro-proliferative effect of Erk1/2 activation (Figure 5), as suggested by earlier studies [27]. We cannot discount involvement of p38 in addition or instead of JNK in such a putative regulatory MAPK cross stalk [28]. While we have not studied here the effect of the DUSP1 overexpression on responses observed in the ocular surface SPSCs [17,18], concurrent declines in pJNK1/2 formation may similarly provide a compensation whereby any negative effect on proliferation caused by reduced pErk1/2 levels is nullified by the enhancement caused by decreased pJNK1/2 (Figure 2B). Additionally, based on the role of activated JNK1/2 on cellular responses to a variety of stress stimuli or pro-inflammatory cytokines, one would expect cells with high constitutive levels of *DUSP1* gene to be better protected than untransduced cells from activation of intracellular pathways leading to apoptosis [29,30] or abnormal differentiation [31].

Finally, the results suggest the physiologic significance of MAPKs-DUSPs stoichiometry on functional outcomes. Knockdown of either *DUSP5* or *DUSP1* fostered higher levels of Erk1/2 phosphorylation, even though the DUSP1 rise in response to EGF was not unaltered. This suggests that the absolute rate of Erk1/2 phosphorylation immediately after addition of EGF and even following the post-activation induction of DUSP protein (Figure 1A-J) expression exceeds the dephosphorylating capacity of each of these two enzymes plus that of DUSP6 and any other phosphatase that may be able to target Erk1/2, e.g., DUSP2 and DUSP4, the two additional classical nuclear phosphatases expressed in the svHCECs. Hence, it will be important to pursue studies examining whether the simultaneous overexpression of two phosphatases, as observed in the SPSC, can cause larger inhibitory effects than those observed in this study with single DUSP6 overexpression.
